# Early response and safety of lenvatinib for patients with advanced hepatocellular carcinoma in a real‐world setting

**DOI:** 10.1002/jgh3.12209

**Published:** 2019-06-10

**Authors:** Takuya Sho, Goki Suda, Koji Ogawa, Megumi Kimura, Tomoe Shimazaki, Osamu Maehara, Taku Shigesawa, Kazuharu Suzuki, Akihisa Nakamura, Masatsugu Ohara, Machiko Umemura, Naoki Kawagishi, Mitsuteru Natsuizaka, Masato Nakai, Kenichi Morikawa, Ken Furuya, Masaru Baba, Yoshiya Yamamoto, Tomoe Kobayashi, Takashi Meguro, Akiyoshi Saga, Takuto Miyagishima, Hideki Yokoo, Toshiya Kamiyama, Akinobu Taketomi, Naoya Sakamoto

**Affiliations:** ^1^ Department of Gastroenterology and Hepatology, Graduate School of Medicine Hokkaido University Sapporo Japan; ^2^ Department of Gastroenterology and Hepatology Japan Community Health Care Organization, Hokkaido Hospital Sapporo Japan; ^3^ Hakodate City Hospital Hakodate Japan; ^4^ Tomakomai City Hospital Tomakomai Japan; ^5^ Hokkaido Gastroenterology Hospital Sapporo Japan; ^6^ Kaisei Hospital Sapporo Japan; ^7^ Kushiro Rosai Hospital Kushiro Japan; ^8^ Hokkaido University, Gastroenterological Surgery 1 Sapporo, Graduate School of Medicine Hokkaido University Sapporo Japan

**Keywords:** early response, lenvatinib, real world, REFLECT

## Abstract

**Background and Aim:**

Lenvatinib has been recently approved as a first‐line systematic therapy for patients with advanced hepatocellular carcinoma (HCC) based on the results of the phase 3 clinical trial REFLECT. This trial excluded patients with a history of systemic chemotherapy, bile duct invasion, and Child‐Pugh grade B. We aimed to investigate the efficacy and safety of lenvatinib for these patients and in the real‐world setting.

**Methods:**

Among patients who were administered lenvatinib for advanced HCC between April and October 2018 in Hokkaido University Hospital and related hospitals, we evaluated those who were followed for more than 2 months and whose treatment response was evaluated via dynamic computed tomography at baseline and 2 months after treatment initiation. Meanwhile, patients were excluded if they had decompensated liver cirrhosis, were followed up less than 2 months, or were not evaluated at 2 months. Patients were also stratified according to compliance with the REFLECT inclusion criteria for further analysis.

**Results:**

A total of 41 patients were included; more than 50% did not meet the REFLECT inclusion criteria. In total, 5 (12.2%), 20 (48.8%), 12 (29.3%), and 4 (9.3%) showed complete response, partial response, stable disease, and progressive disease, respectively. The objective response rate was 61.2%. The objective response rate and disease control rate were similar between patients who did and did not meet the REFLECT inclusion criteria. Moreover, the safety profile was also similar between the two patient groups.

**Conclusion:**

Lenvatinib showed high early response rate and tolerability in patients with advanced HCC. Favorable outcomes were similarly observed in patients who did not meet the REFLECT inclusion criteria.

## Graphical abstract

Lenvatinib has been recently approved for patients with advanced hepatocellular carcinoma based on the results of the REFLECT trial, which excluded patients with a history of systemic chemotherapy, bile duct invasion, and Child‐Pugh grade B. We showed the efficacy and safety of lenvatinib for those patients and also for patients who did not meet the REFLECT inclusion criteria in a real‐world setting.

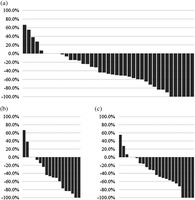

## Introduction

Hepatocellular carcinoma (HCC) is the third leading cause of cancer‐related death worldwide and is thus an important health concern.^1^ Despite oncological advances, the prognosis of patients with advanced HCC has remained poor,^2^, ^3^, ^4^ partly because of the limited therapeutic options available for this malignancy. The optimal treatment strategy for HCC is a multimodal approach that includes multikinase inhibitors. Sorafenib is the first multikinase inhibitor approved for advanced HCC, and its capability to prolong overall survival (OS) and time to progression in patients with advanced HCC was first reported by the SHARP trial.^5^ Until recently, the available systemic treatments for patients with advanced HCC were limited to sorafenib because various clinical trials failed to show any significant efficacy of novel systemic treatments for patients with advanced HCC or noninferiority of novel systemic treatments to the current standard therapy of sorafenib.^6^, ^7^, ^8^ Recently, regorafenib, a multikinase inhibitor, was approved as second‐line systemic therapy for patients with advanced HCC who failed sorafenib therapy.^9^ Lenvatinib, a novel multikinase inhibitor, has also been recently approved as a first‐line systematic therapy for patients with advanced HCC. The phase 3 clinical trial REFLECT^10^ was the first to show that the OS of patients with advanced HCC who were treated with lenvatinib is noninferior to that of patients treated with sorafenib. In addition, the progression‐free survival of patients treated with lenvatinib was significantly longer than that of patients treated with sorafenib. However, in the REFLECT trial, patients who were treated with another multikinase inhibitor (sorafenib and/or regorafenib), had an HCC occupying ≥50% of the liver, had obvious invasion of the bile duct, demonstrated invasion at the main portal vein, had a Child‐Pugh grade B, and had hemoglobin <8.5 g/dL or platelet count <75 × 10^9^/L were excluded. Thus, the safety and efficacy of lenvatinib for such patients are not clarified. In addition, real‐world data are also limited.

Therefore, this study aimed to evaluate the early therapeutic response to lenvatinib in patients with nonresectable HCC in the real‐world setting, focusing on patients who did not meet the inclusion criteria of the REFLECT trial but did not have a contraindication according to the package insert of lenvatinib (Lenvima Capsules, Eisai Co., Ltd., Tokyo, Japan).

## Methods

### *Patients*

This was a retrospective multicenter study that enrolled patients who were given lenvatinib for advanced HCC between April and October 2018. The inclusion criteria were: (i) meeting the diagnostic criteria for advanced HCC according to the American Association for the Study of Liver Diseases guidelines,^11^ (ii) follow up for more than 2 months after treatment initiation, (iii) treatment response was evaluated via dynamic computed tomography (CT) at baseline and 2 months after treatment initiation, and (iv) having adequate clinical data. Meanwhile, patients were excluded if they (i) had decompensated liver cirrhosis, (ii) were followed up less than 2 months, (iii) were treated with drugs listed in the contraindications for coadministration in the package insert of lenvatinib, and (iv) were not evaluated for treatment response at 2 months after treatment initiation.

We collected data on gender, age, etiology, blood cell count, alpha‐fetoprotein (AFP), des‐gamma‐carboxyprothrombin, the number of hepatic lesions and their maximum diameter, Child‐Pugh score, albumin‐bilirubin (ALBI) grade, and Barcelona Clinic Liver Cancer (BCLC) stage at baseline. Patients were assessed using laboratory tests and physical findings minimally at 2, 4, 6, and 8 weeks after treatment initiation to evaluate treatment response and safety. In addition, the efficacy and safety of lenvatinib for advanced HCC was evaluated among patients who did and did not meet the REFLECT trial inclusion criteria.

This study conformed to the ethical guidelines of the Declaration of Helsinki and was approved by the ethics committees of Hokkaido University Hospital (approval no. 017‐0521) and participating institutions. Informed consent was obtained from all patients.

### *Treatment protocol*

Lenvatinib (Lenvima) was administered orally for advanced HCC. The lenvatinib dose depended on the patients’ weight: those who weighed <60 kg were administered 8 mg of lenvatinib once daily, while those who weighed ≥60 kg were initially administered 12 mg of lenvatinib once daily. However, patients with Child‐Pugh grade B were initially treated with 8 mg of lenvatinib once daily regardless of weight.

Lenvatinib was discontinued when unacceptable adverse events (AEs) or disease progression was observed. In addition, the lenvatinib dose was adjusted, or treatment was interrupted, if the patients developed grade ≥3 or unacceptable AEs until the symptom resolved, as indicated on the package insert. AEs were evaluated according to the National Cancer Institute Common Terminology Criteria for Adverse Events, version 4.0.

### *Evaluation of treatment response*

Dynamic CT was performed at baseline and 8 weeks after treatment initiation to evaluate treatment response. The responses were classified by the attending physician according to the modified Response Evaluation Criteria in Solid Tumors.^12^ Complete response was defined as the disappearance of all evidence of disease. Partial response was defined as a decrease of at least 30% in the sum of the longest diameters of the target lesions without the appearance of any new lesions. Progressive disease was defined as an increase of at least 20% in the sum of the longest diameters of the target lesions in the liver or the appearance of new lesions. Stable disease was defined as not meeting the criteria for complete response, partial response, or progressive disease. The efficacy of lenvatinib was further evaluated among patients who did and did not meet the REFLECT trial inclusion criteria.

### *Statistical analysis*

Continuous variables were analyzed using the paired Mann–Whitney *U* test, while categorical variables were analyzed using the chi‐square test and Fisher's exact test. Statistical analyses were performed using SPSS Statistics 22.0 (IBM Corp., Armonk, NY, USA), and *P* < 0.05 was considered statistically significant.

## Results

### *Patient characteristics*

**Figure 1:**
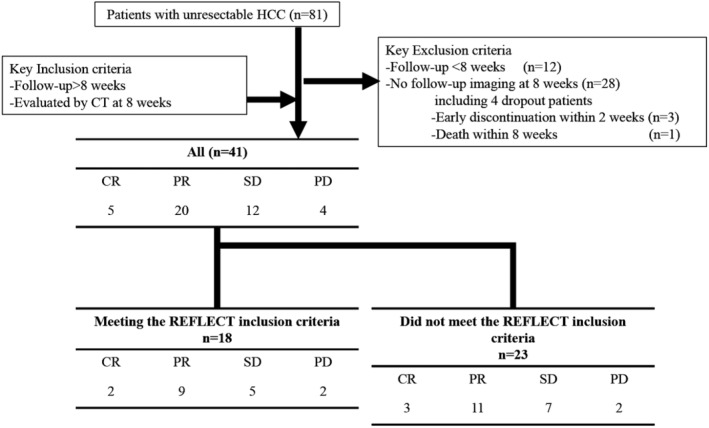
Study flowchart. CR, complete response; CT, computed tomography; HCC, hepatocellular carcinoma; PD, progressive disease; PR, partial response; SD, stable disease.

Between April 2018 and October 2018, a total of 81 patients were started on lenvatinib for advanced HCC. Of these, 40 patients were excluded because they were followed up for less than 2 months (*n* = 12) or did not undergo CT examination at 2 months after treatment initiation (*n* = 28). In the 28 patients who did not undergo CT examination at 2 months after treatment initiation, 3 patients who discontinued lenvatinib within 2 weeks due to AEs and 1 patient who died within 2 months were included. Thus, 41 patients were enrolled in this study (Fig. 1). The baseline patient characteristics are shown in Table 1. The median patient age was 71 years (range, 46–97 years), and 37 (90.2%) patients were men. Fourteen patients were infected with hepatitis B virus, and seven patients were infected with the hepatitis C virus. The others had non‐B, non‐C etiology (*n* = 20). The most common Child‐Pugh score was 5 (*n* = 22), followed by a score of 6 (*n* = 14). Meanwhile, five patients had a Child‐Pugh score of more than 6. A majority of patients had ALBI grade 2 (*n* = 29, 70.7%) and BCLC stage C (*n* = 27, 65.9%. Ten patients had extrahepatic metastases. The median serum AFP level was 15.4 IU/mL (range, 1.6–449 909.0 IU/mL). There were 23 (56.1%) patients who did not meet the REFLECT inclusion criteria (history of tyrosine kinase inhibitor [TKI], *n* = 16; Child‐Pugh score B, *n* = 5; reduced platelet count, *n* = 2; bile duct invasion, *n* = 4; and performance status score 2, *n* = 1). These patients had a significantly higher AFP level (*P* = 0.044) and a higher Child‐Pugh score (*P* = 0.0165) (Table 1).

**Table 1:** Baseline patient characteristics

Clinical characteristics	Overall cohort (*n* = 41)	Met the REFLECT criteria (*n* = 18)	Did not meet the REFLECT criteria (*n* = 23)	*P* value
Age (years)	71 (46–97)	75 (46–83)	70 (54–97)	0.1026
Gender				0.0259
Male	37	18	19	
Female	4	0	4	
Etiology				0.0311
HBV	14	3	11	
HCV	7	2	5	
Others	20	13	7	
ECOG PS				0.3003
0	28	11	17	
1	12	7	5	
2	1	0	1	
BMI (kg/m^2^)	23.9 (13.5–33.4)	24.4 (13.5–29.7)	22.7 (17.4–33.4)	0.4863
White blood cell (/mm^3)^	4600 (2000–9900)	4500 (2900–9100)	4700 (2000–9900)	0.8954
Neutrophil (/mm^3^)	2645 (1360–5788)	2663 (1705–5788)	2501 (1360–5379)	0.6303
Neutrophil/lymphocyte ratio	2.79 (0.85–9.00)	2.41 (0.97–9.00)	3.62 (0.85–5.23)	0.2752
Platelet (×10^4^/μL)	13.8 (4.4–33.6)	14.7 (8.5–25.8)	13.6 (4.4–33.6)	0.6176
Prothrombin time (%)	94.0 (46.6–150.0)	98.5 (74.3–150.0)	88.3 (46.6–116.9)	0.0978
NH3 (μg/dL)	41 (13–118)	35 (18–76)	43 (13–118)	0.1565
Albumin (g/dL)	3.7 (2.8–4.5)	3.8 (3.0–4.5)	3.5 (2.8–4.3)	0.0615
Total bilirubin (mg/dL)	0.7 (0.2–3.1)	0.7 (0.2–2.1)	0.7 (0.3–3.1)	0.6535
ALBI grade				0.6135
1	12	6	6	
2	29	12	17	
AST (IU/L)	37 (19–181)	32 (23–93)	38 (19–118)	0.5281
ALT (IU/L)	23 (13–96)	24 (13–96)	23 (13–96)	0.9266
Child‐Pugh score				0.0165
5	22	13	9	
6	14	5	9	
7–9	5	0	5	
AFP (ng/mL)	15.4 (1.6–449 909.0)	5.8 (2.0–19 394.3)	52.3 (1.6–449 909.0)	0.0444
DCP (mAU/mL)	734 (12–43 200)	384 (15043200)	1409 (13–27 425)	0.1458
Maximum intrahepatic tumor size (mm)	37 (8–135)	41 (10–123)	36 (8–135)	0.6254
Number of intrahepatic tumors				0.4371
None	3	6	1	
1	11	10	5	
Multiple	27	2	17	
TNM stage				0.8603
II	3	2	1	
III	17	7	10	
IVA	11	5	6	
VIB	10	4	6	
BCLC stage				
B	14	6	8	
C	27	12	15	0.9226
Met the Milan criteria	2 (4.9%)	1 (5.6%)	1 (4.3%)	0.8591
Positive for Vp	10 (24.4%)	4 (22.2%)	6 (26.1%)	0.8532
Vp2	4	2	2	
Vp3	6	2	4	
Vp4	0	0	0	
Positive for Vv	2 (4.9%)	2 (11.1%)	0 (0%)	0.0642
Positive for bile duct invasion	4 (9.8%)	0 (0%)	4 (17.4%)	0.0259
Positive for LN metastasis	5 (12.2%)	2 (11.1%)	3 (13.0%)	0.8507
Positive for EHM	10 (24.4%)	4 (22.2%)	6 (26.1%)	0.7743
Naïve: recurrence	7:36	3:15	2:21	0.1500
History of hypertension	25 (61.0%)	10 (55.6%)	15 (65.2%)	0.5294
History of hepatectomy	16 (39.0%)	6 (33.3%)	10 (43.5%)	0.5074
History of RFA	11 (26.8%)	5 (27.8%)	6 (26.1%)	0.9036
History of TACE	30 (73.2%)	13 (72.2%)	17 (73.9%)	0.9036
History of sorafenib	16 (39.0%)	0 (0%)	16 (69.6%)	<0.0001
History of regorafenib	4 (9.8%)	0 (0%)	4 (17.4%)	0.0259

Data are presented as median (range) or in *n*.

AFP, alpha‐fetoprotein; ALBI grade, albumin‐bilirubin grade; ALT, alanine aminotransferase; AST, aspartate transaminase; BCLC, the Barcelona Clinic Liver Cancer; BMI, body mass index; DCP, des‐gamma‐carboxy prothrombin; ECOG PS, Eastern Cooperative Oncology Group performance status; EHM, extra‐hepatic metastasis; HBV, hepatitis B virus; HCV, hepatitis C virus; LN, lymph node; RFA, radiofrequency ablation; TACE, transcatheter arterial chemoembolization; TNM, tumor node metastasis stage of the Liver Cancer Study Group of Japan; Vp, portal vein invasion; Vv, hepatic vein invasion.

### *Treatment response*

Treatment response at 8 weeks after treatment initiation was evaluated in all patients. Of the 41 patients, 5 (12.2%), 20 (48.8%), 12 (29.3%), and 4 (9.3%) showed complete response, partial response, stable disease, and progressive disease, respectively (Table 2). The objective response rate (i.e. the total rate of patients with complete response and partial response) was 61.2%. The disease control rate (i.e. the total rate of patients with complete response, partial response, and stable disease) was 90.2%. Among the patients who did not meet the REFLECT inclusion criteria, the objective response rate was 56.3% (9/16), 60% (3/5), and 100% (4/4) in those with a history of TKI administration, Child‐Pugh score B, and bile duct invasion, respectively.

**Table 2:** Clinical response to lenvatinib

Response	Overall cohort (*n* = 41)	Met the REFLECT criteria (*n* = 18)	Did not meet the REFLECT criteria (*n* = 23)	*P* value
Complete response, *n* (%)	5 (12.2)	2 (11.1)	3 (13.0)	
Partial response, *n* (%)	20 (48.8)	9 (50.0)	11 (47.8)	
Stable disease, *n* (%)	12 (29.3)	5 (27.8)	7 (30.4)	
Progressive disease, *n* (%)	4 (9.8)	2 (11.1)	2 (8.7)	
Objective response rate	61.0% (25/41)	61.1% (11/18)	60.9% (14/23)	0.8293
Disease control rate	90.2% (37/41)	88.9% (16/18)	91.3% (21/23)	0.7965

**Figure 2:**
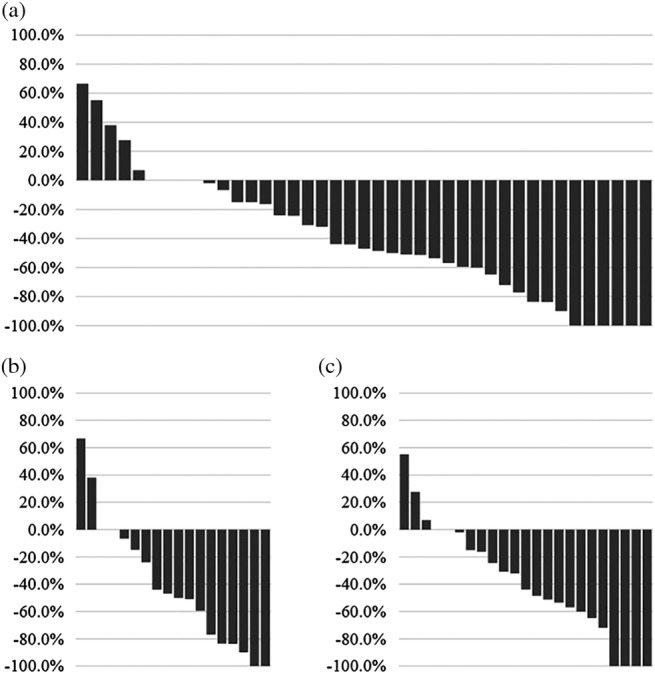
Waterfall plot of changes in targeted tumor size as assessed according to mRECIST in the (a) overall patient cohort; (b) patients who meet the REFLECT inclusion criteria and (c) patients who did not meet the REFLECT inclusion criteria.

The objective response rate (*P* = 0.8293) and disease control rate (*P* = 0.7965) were similar between patients who did and did not meet the REFLECT inclusion criteria. Moreover, the tumor reduction ratio (Fig. 2) and rate of AFP change were also similar between the two patient groups (*P* = 0.8849 and *P* = 0.7743).

### *Safety and treatment discontinuation due to AEs*

The safety profile of lenvatinib as assessed between patients who did and did not meet the REFLECT inclusion criteria is summarized in Table 3. Overall, the most common AEs of any grade were hand‐foot syndrome (*n* = 23, 56.1%), general fatigue (*n* = 24, 58.5%), loss of appetite (*n* = 28, 68.3%), hypertension (*n* = 28, 68.3%), and increased urinary albumin (*n* = 23, 56.1%). Meanwhile, the most common grade >3 AEs were hand‐foot syndrome (*n* = 6, 14.6%), hypertension (*n* = 5, 12.2%), and decreased platelet count (*n* = 5, 12.2%). The rate of grade >3 AEs was similar between patients who did and did not meet the REFLECT inclusion criteria**.**

**Table 3:** Adverse events and treatment discontinuations

	Overall cohort (*n* = 41)	Met the REFLECT criteria (*n* = 18)	Did not meet the REFLECT criteria (*n* = 23)	*P* value
Treatment discontinuation	3 (7.3%)	1 (5.6%)	2 (8.7%)	0.6982
Interruption and/or dose reduction	30 (73.2%)	15 (83.3%)	15 (65.2%)	0.186
Worsened Child Pugh score	18 (43.4%)	8 (44.4%)	10 (43.5%)	0.9507

Adverse events	Any grade	Grade > 3	Any grade	Grade > 3	Any grade	Grade > 3
HFS	23 (56.1%)	6 (14.6%)	12 (66.7%)	3 (16.7%)	11 (47.8%)	3 (13.0%)
General fatigue	24 (58.5%)	0 (0%)	13 (72.2%)	0 (0%)	11 (47.8%)	0 (0%)
Appetite loss	28 (68.3%)	1 (2.4%)	14 (77.8%)	1 (5.6%)	14 (60.9%)	0 (0%)
Diarrhea	9 (22.0%)	1 (2.4%)	3 (16.7%)	1 (5.6%)	6 (26.1%)	0 (0%)
Hypertension	28 (68.3%)	5 (12.2%)	15 (83.3%)	2 (11.1%)	13 (56.5%)	3 (13.3%)
Hepatic coma	3 (7.3%)	3 (7.3%)	1 (5.6%)	1 (5.6%)	2 (8.7%)	2 (8.7%)
Weight loss	6 (14.6%)	0 (0%)	5 (27.8%)	0 (0%)	1 (4.3%)	0 (0%)
Proteinuria	23 (56.1%)	1 (2.4%)	11 (61.1%)	1 (5.6%)	12 (52.2%)	0 (0%)
Decreased platelet count	21 (51.2%)	5 (12.2%)	10 (55.6%)	2 (11.1%)	11 (47.8%)	3 (13.0%)
Fever	1 (2.4%)	0 (0%)	0 (0%)	0 (0%)	1 (4.3%)	0 (0%)
Hypothyroidism	21 (51.2%)	0 (0%)	11 (61.1%)	0 (0%)	10 (43.5%)	0 (0%)
Dysgeusia	3 (7.3%)	0 (0%)	1 (5.6%)	0 (0%)	2 (8.7%)	0 (0%)
Rash	1 (2.4%)	1 (2.4%)	1 (5.6%)	1 (5.6%)	0 (0%)	0 (0%)
Decreased albumin	21 (51.2%)	0 (0%)	9 (50.0%)	0 (0%)	12 (52.2%)	0 (0%)
Increased bilirubin	8 (19.5%)	1 (2.4%)	4 (22.2%)	1 (5.6%)	4 (17.4%)	1 (4.3%)
Ascites	5 (12.2%)	0 (0%)	1 (5.6%)	0 (0%)	4 (17.4%)	0 (0%)
Hyperthyroidism	1 (2.4%)	0 (0%)	1 (5.6%)	0 (0%)	0 (0%)	0 (0%)
Stomatitis	1 (2.4%)	0 (0%)	1 (5.6%)	0 (0%)	0 (0%)	0 (0%)
Increased creatinine	4 (9.8%)	0 (0%)	2 (11.1%)	0 (0%)	2 (8.7%)	0 (0%)

Data are presented as *n* (%).

HFS, hand‐foot syndrome.

Overall, three patients (7.3%) discontinued treatment due to drug‐related AEs (hyperbilirubinemia, *n* = 1; hepatic encephalopathy, *n* = 2). In addition, treatment was interrupted or the dose was reduced in 30 patients (73.2%). The rate of treatment discontinuation and treatment interruption and/or dose reduction was similar between patients who did and did not meet the REFLECT inclusion criteria.

Next, we evaluated the changes in Child‐Pugh score between baseline and at 8 weeks after lenvatinib initiation. Eighteen patients (43.4%) had a worsened Child‐Pugh score (Table 3). The rate of worsened Child‐Pugh score was similar between patients who did and did not meet the REFLECT inclusion criteria. However, the rate of worsened Child‐Pugh score was significantly higher among patients with a Child‐Pugh score of ≥6 (*n* = 19) than that of patients with a Child‐Pugh score of 5 (*n* = 22) (12/19 (63.2%) *vs* 6/22 (27.3%), *P* = 0.019).

## Discussion

In this real‐world retrospective multicenter study of lenvatinib for patients with advanced HCC, more than 50% of the included patients did not meet the REFLECT trial inclusion criteria. Overall, the early response and tolerability were favorable and were similar between patients who did and did not meet the REFLECT trial inclusion trial. Thus, lenvatinib might be safe and effective even for patients who did not meet the REFLECT inclusion criteria.

The multikinase inhibitor sorafenib has only been approved as first‐line systemic therapy for patients with advanced HCC for almost 10 years. Although the recently concluded phase 3 trial REFLECT showed the noninferiority of OS in lenvatinib compared with that in sorafenib for patients with advanced HCC,^10^ the trial excluded patients with bile duct invasion, Child‐Pugh grade B, and reduced platelet or hemoglobin count. Thus, the efficacy and safety of lenvatinib for these patients have not been clarified. In the current real‐world study, more than 50% of patients started on lenvatinib did not meet the REFLECT trial inclusion trial. This helped to clarify the efficacy and safety of lenvatinib for these patients.

Lenvatinib is an orally active TKI targeting VEGFR1–3, FGFR1–4, PDGFR‐α, c‐Kit, and RET.^13^, ^14^ Thus, compared with sorafenib, lenvatinib could inhibit several additional cell signalings, including fibroblast growth factor (FGF) signaling. Recently, in vitro and in vivo analyses demonstrated that acquired resistance to sorafenib posttreatment is mediated by the activation of FGF signaling.^15^, ^16^ Thus, lenvatinib might be effective for patients who previously failed to respond to sorafenib because lenvatinib could inhibit FGF signaling. Similar to the results of the current study, the study by Hiraoka *et al*.^17^ also showed favorable treatment outcomes of lenvatinib for patients who had a history of TKI, supporting the results of the in vitro and vivo analyses. However, these findings still need to be validated in further studies with large a sample size.

In this study, the objective response rate was better than that of the REFLECT trial. Notably, a subgroup analysis of Japanese patients in the REFLECT study^18^ showed a higher objective response rate, thus indicating that race might affect the treatment outcomes of lenvatinib. A total of 78% (32/41) of patients in the current study had a baseline AFP level of <200 ng/mL. It is reported that baseline AFP level affected the prognosis and treatment outcomes^10^ of patients treated with systemic chemotherapy for HCC.^19^ In addition, the number of patients with extrahepatic metastasis, which predicts poor response,^10^ was lower than that of the REFLECT trial. Thus, the high number of patients with lower baseline AFP level and small number of patients with extrahepatic metastasis in the current study might have affected the favorable outcomes obtained. In addition, the limited number of included patients might have also affected the treatment outcome.

In this study, we analyzed the early response to and safety of lenvatinib for patients with advanced HCC at 8 weeks after treatment initiation. Therefore, the results could not show the patients’ prognosis. However, Lencioni *et al*. recently reported that objective response and OS were significantly correlated in systemic therapy for patients with advanced HCC.^20^ The objective response rate of lenvatinib in this study seemed to be favorable compared with previously reported outcomes on sorafenib.^5^ Thus, the favorable efficacy of lenvatinib for patients who did not meet the REFLECT criteria might predict favorable prognosis. In addition, Kudo *et al*.^18^ also reported that this favorable response rate might motivate patients, resulting in higher compliance.

Three patients (7.3%) discontinued lenvatinib due to drug‐related AEs, and treatment was interrupted or the dose was reduced in 30 patients (73.2%). However, as shown in Table 3, the occurrence rate of these events was similar between patients who did and did not meet the REFLECT inclusion criteria. This finding indicates that lenvatinib is safe and tolerable even in patients who did not meet the REFLECT inclusion criteria.

The most common any‐grade AEs were similar between the current study and those in REFLECT and included hand‐foot syndrome, general fatigue, appetite loss, and hypertension. Most AEs were controllable. However, two patients discontinued lenvatinib due to hepatic encephalopathy. Both patients had esophageal varices at baseline. Thus, patients with portal hypertension at baseline should be monitored closely for hepatic encephalopathy during treatment.

Overall, 43.4% (18/41) of patients had a worsened Child‐Pugh score (Table 3). The occurrence rate of worsened Child‐Pugh score was similar between patients who did and did not meet the REFLECT inclusion criteria. However, the rate of worsened Child‐Pugh score was significantly higher in patients with a baseline Child‐Pugh score of ≥6 than those with a score of 5 (63.2*vs* 27.3%, *P* = 0.019). Because the prognosis of patients with HCC is significantly affected by hepatic function,^21^ lenvatinib therapy yields more benefit when initiated, while hepatic function is still preserved.

This study has several limitations that must be considered when interpreting the results. First, the study was retrospective in design; included a limited number of patients; and had several missing data, including PT‐INR and MELD scores. The observation period was also short and limited to only 8 weeks. Moreover, we included three patients who discontinued lenvatinib within 2 weeks due to AEs and one patient who died within 2 months in the group of 28 excluded patients who did not undergo CT examination at 2 months after treatment initiations. In addition, the patients who did not meet the REFLECT inclusion trial were heterogeneous. Therefore, prospective studies with larger cohorts and longer observation periods are needed to validate our findings.

In conclusion, this real‐world study showed that lenvatinib yields a high early response rate and tolerability for advanced HCC in both patients who did and did not meet the REFLECT trial inclusion criteria.
